# Investigation of Residual Stresses in Extruded Thermoplastic PE-RT Pipes

**DOI:** 10.3390/polym18091135

**Published:** 2026-05-05

**Authors:** Arun Biradar, Pierre Mertiny

**Affiliations:** Department of Mechanical Engineering, University of Alberta, 9211-116 St, Edmonton, AB T6G 1H9, Canada; abiradar@ualberta.ca

**Keywords:** polymer pipes, PE-RT, residual stress, annealing, crystallinity

## Abstract

Polyethylene of Raised Temperature resistance (PE-RT) is a versatile polymer with an enhanced ability to withstand higher temperatures than other standard polyethylene-derived materials. The application scope of PE-RT pipes extends across a broad range of residential and industrial uses. However, the inherent residual stresses developed during the fabrication processes of these pipes pose a significant challenge to their service performance. Moreover, the technical literature on residual stresses in PE-RT pipes is scarce. The investigations presented in this paper address this scientific and technical gap by experimentally quantifying the residual stresses present in different sizes of PE-RT pipes manufactured at the same plant. The slitting method was employed as the primary experimental procedure in these investigations. In addition to establishing experimental processes, this study aims to mitigate residual stresses in PE-RT pipes by investigating their sensitivity to varying annealing temperatures for one selected pipe size. Subsequently, the optimal annealing temperature (115 °C) was applied to the remaining pipe sizes to determine its efficacy in residual stress mitigation. Through the implementation of these procedures, the circumferential residual stresses in PE-RT pipes subjected to annealing at 115 °C were significantly reduced, with a minimum decrease of 78%. Furthermore, the longitudinal residual stresses were substantially diminished, reaching a state of near-complete elimination.

## 1. Introduction

Pipes and similar components like liners fabricated using polyethylene of raised temperature resistance (PE-RT) have gained significant traction in various residential and industrial piping systems due to their flexibility, durability, and advanced chemical and thermal performance compared to several other grades of polyethylene. The evolution of PE-RT pipes originates from their unique chemical composition involving copolymerization of ethylene with commonly used α-olefins such as propylene, 1-butene, 1-hexene, and 1-octene, resulting in improved long-term mechanical stability and high-temperature and pressure resistance, in turn enhancing the inherent flexibility of these pipes for various installations [1]. A notable example that highlights the advantages of PE-RT pipes was reported for the Canfor Prince George Pulp & Paper Mill in BC, Canada [2]. This plant faced repeated failures in underground fiberglass reinforced pipe (FRP) used for a bleach plant effluent system, posing significant concern for environment and operational reliability. In just over five years, the FRP system needed more than ten repairs. With the operation temperatures reaching up to 75 °C, conventional PE pipes were not suitable. To overcome this issue, the mill replaced the aging FRP system with a PE-RT pipe system, expanding the use of PE pipes into larger diameter industrial applications and allowing operating temperatures up to 82 °C with intermittent temperatures reaching up to 95 °C. It was reported that many industries have incorporated PE-RT pipes into their products in recent years.

Appreciating the versatile nature of PE-RT, most of the research conducted on corresponding pipes has been on examining long-term performance, particularly in terms of creep characteristics, oxidative degradation, impact resistance when buried, and susceptibility to hydrothermal or thermal aging [3,4,5]. Residual stresses in polyethylene pipes have also been recognized as an important factor affecting structural integrity, fracture behavior, and service lifetime. For example, Guevara-Morales and Leevers observed that if residual stresses in polyethylene pipes are not managed properly, significantly accelerated rapid crack propagation may ensue by affecting the strain energy stored and released during crack propagation [6]. Poduška et al. [7] investigated residual stresses in PE pipes using a 13-ring layer-removal procedure for hoop-stress evaluation together with sectioning- and modeling-assisted assessment of axial residual stress, while Hutař et al. [8] highlighted the significance of residual stresses in relation to polymer-pipe lifetime. Therefore, residual stresses in PE pipes cannot be regarded as an unexplored subject in general. However, the available literature remains limited in the specific context of extruded PE-RT pipes, particularly for a systematic experimental investigation that simultaneously addresses different pipe sizes, annealing effects, and crystallinity variations. Along with crack propagation, the effects of residual stresses include reductions in fatigue strength and stress corrosion cracking resistance. Additionally, when interacting with elevated service temperatures, these stresses can significantly reduce the operational reliability of PE-RT pipes and lead to compromised structural integrity and premature failure [9]. Therefore, the need exists to quantify, analyze, and comprehend the presence of residual stresses in widely used extruded PE-RT pipes, particularly within the narrower scope addressed in the present study.

Residual stresses can be assessed using various methods, including X-ray diffraction, hole drilling, and slitting methods [10,11,12]. The authors would like to point out that quantifying residual stresses in polymer pipes is still an evolving research field. Additionally, the aforementioned methods have been attempted using pipes made of various polymers, such as polypropylene, PE and polyamide. In the present study, a slitting-based approach was selected because it provides a practical and mechanically realizable experimental route for assessing residual stresses in thin-walled PE-RT pipes. Compared with the 13-ring layer-removal procedure reported by Poduška et al. [7], the present slitting-based method requires substantially less specimen preparation and machining, since it relies only on vertical slits to remove a section rather than sequential ring preparation and progressive layer removal. This makes the present approach more straightforward to implement for systematic testing of thin-walled PE-RT pipes across multiple pipe sizes and annealed conditions. In addition, this approach enabled identification of a suitable specimen aspect ratio for reliable circumferential residual-stress assessment. Therefore, in the present context, the slitting method was employed as an effective quantification technique for identifying residual stresses in PE-RT pipes.

Residual stresses arise in pipes due to cooling conditions imposed during their extrusion process. All three sizes of PE-RT pipes investigated here are extruded in a similar manufacturing environment at the same plant, i.e., these pipes are quenched with cold water, which is sprayed only on the outer portion of the pipes immediately after extrusion, while the inner portion remains at higher temperature from extrusion. This allows for the development of a temperature gradient and hence the development of residual stresses, specifically, compressive stresses on the outer layer and tensile stresses on the inner layer. If these pipes were cooled in a similar manner on both outer and inner portions, compressive stresses would be present on both outer and inner portions with tensile stresses along the central wall portion [13]. The presence of tensile residual stresses on the inner surface and compressive stresses on the outer surface of extruded PE-RT pipes can exacerbate crack growth due to the combined effect of internal pressure and residual tensile stresses during service [14]. Hence, the present investigation of residual stresses in PE-RT pipes makes an important contribution to the design and fabrication of such structures, in particular by providing quantitative measures for their assessment.

In general, residual stresses are considered undesirable for the optimal performance of PE-RT pipes. Consequently, the sensitivity of these residual stresses to heat treatment is also investigated with the objective of mitigating them. Therefore, the present study also investigates the effect of annealing on residual stresses along with evaluating crystallinity of samples. Annealing is well known as a crucial heat treatment process employed to obtain better molecular structure through recrystallization. Consequently, significant enhancements in performance may ensue in extruded PE-RT pipe and other semi-crystalline polymers. Annealing temperature and time must be carefully selected to allow for the polymer molecular structure to undergo beneficial changes, such as increasing crystallinity and improving mechanical properties. The three common mechanisms occurring during annealing are molecular relaxation, realignment of molecular chains or recrystallization, and microstructural changes such as variation in lamellar thickness, molecular orientation and phase changes [15,16,17]. Loos and Tian [18,19] showed that single polyethylene crystals can undergo significant recrystallization at temperatures close to their melting point, ultimately enhancing their crystalline arrangements and leading to improved mechanical properties. It should be noted that the implementation of a heat treatment process after extrusion in the manufacturing plant is of economical concern, given additional energy, storage, and maintenance needs, and thus costs, for annealed pipes that are often stored as large spools.

The objective of this study is to perform a systematic investigation to quantify the residual stresses developed during extrusion of three different sizes of PE-RT pipes. Particular focus is placed on applying and assessing the slitting method as an experimental technique for quantifying residual stresses in both the circumferential and longitudinal directions for extruded PE-RT pipes of different sizes. Furthermore, the sensitivity of these residual stresses to elevated annealing temperatures is investigated, together with their possible correlation to variations in crystallinity. The central hypothesis of this study is that rapid quenching of the outer pipe surface during extrusion leads to differences in polymer morphology between the outer and inner pipe portions, resulting in the development of residual stresses. It is further hypothesized that this mechanism manifests as lower crystallinity at the outer wall compared with the inner wall, thereby creating a crystallinity gradient across the pipe thickness. These residual stresses, together with the crystallinity gradient, are hypothesized to be mitigated through annealing. To the authors’ knowledge, systematic experimental quantification of residual stresses in extruded PE-RT pipes of different diameters and their mitigation through annealing has not been previously reported in the literature.

## 2. Materials and Methods

This investigation was performed using PE-RT pipes supplied by a single manufacturer (Flexpipe, Calgary, AB, Canada). Different nominal pipe sizes were selected to elucidate how residual stresses vary with diameter in pipes extruded from the same material. Specifically, pipes with three nominal diameters of 3-, 4- and 6-inch were used to study residual stresses and their mitigation. Pipes with a length of 3 m were received from the supplier. Detailed dimensions and other relevant properties for these pipes are presented in Table 1. For instance, the pipe outside diameter (*OD*) was measured at five different angles along the circumference (at 36° intervals) at a specific pipe location. This was repeated for a total of five locations along the pipe length, yielding the average *OD* of the pipe while also determining the ovality of the received pipes at these locations. The wall-thickness of a pipe specimen was measured by averaging the readings at both ends with an accuracy of 0.025 mm.

For assessing the residual stresses in PE-RT pipes, a methodology adapting the ASTM E1928-13 standard was developed [20]. As stipulated in the standard, a linear stress distribution over the thickness of the pipe wall is assumed for thin-walled tubes. As shown in Table 1, the ratio of outer diameter to wall thickness, also known as the Standard Dimension Ratio (SDR), was approximately 17 for all studied pipe sizes. Consequently, the wall thicknesses were well below one-tenth of the outer diameter, and all pipes satisfied the thin-walled condition underlying the standard. Therefore, the assumption of a linear stress distribution was considered appropriate for the present pipes. Although more elaborate techniques may be used to reconstruct a more detailed non-linear through-thickness stress distribution, such methods become experimentally more onerous to implement for thin-walled pipes, especially when multiple pipe sizes and annealed conditions are investigated systematically. In the present context, the linear-distribution assumption provides a practical basis for estimating the residual stress level, while at the same time minimizing potential disturbances of existing stress fields caused by extensive machining operations. Moreover, because the primary objective was to quantify the critical (or maximum) residual stresses rather than to reconstruct the entire through-thickness stress profile in detail, the linear assumption was considered appropriate for the present analysis.

**Table 1:** Specifications of PE-RT pipes used for experimentation.

Nominal Pipe Size	Average Outer Diameter, mm	Average Inner Diameter, mm	Average Wall Thickness, mm	Ovality, %	Melting Point ^1^, °C	Yield Strength ^1^, MPa	SDR ^2^
3-inch	87.38	77.01	5.18	1.5–2.0	132	24.1	16.86
4-inch	112.45	98.98	6.73	2–2.5	132	24.1	16.71
6-inch	161.29	142.29	9.50	2.5–3.0	132	24.1	16.98

^1^ Melting point and yield strength were obtained from the technical data sheet [21]. ^2^ SDR: Ratio of outer diameter to wall thickness of the pipe.

### 2.1. Slitting Method for Quantifying the Residual Stresses

Adhering to the guidelines outlined in the ASTM E1928-13 standard, the tests were conducted on samples cut from adjacent sections of a single specific type of PE-RT pipe, in either the as-delivered or annealed condition. Prior to machining of the pipe specimens, all pertinent dimensions were carefully measured and recorded from the as-received 3-m-long pipe sections using the procedures outlined in the standard.

Multiple specimens with different aspect ratios (*AR*), i.e., length versus outer diameter, were prepared. In earlier work [22], samples with *AR* ranging from 0.2 to 3.0 were used for the investigations of 3-inch pipe, which was repeated in the present work (0.2, 0.4, … 2.0, 2.5, 3.0 for a total of 12 *AR* values). The results indicated that the maximum circumferential residual stress exhibited minimal sensitivity to *AR* for *AR* ≳ 1.4, suggesting that the number of tests can be reduced in this range, and that increasing *AR* beyond 2.0 provides no meaningful additional benefit for capturing the peak residual stress response. Accordingly, *AR* values greater than 2.0 were excluded in the present investigation of 6-inch pipes.

The previous study also suggested that specimens with *AR* < 1.0 produced lower values and did not approach the observed maximum circumferential residual stress. To assess whether this behavior is pipe size dependent, experiments on 6-inch pipe were performed using *AR* ranging from 0.2 to 2.0 (0.2, 0.4, … 2.0 for a total of ten *AR* values). Similar to the previous work, the results showed that *AR* of 1.4 and higher resulted in stable estimates of circumferential residual stress, while *AR* of 0.2 to 1.0 consistently produced lower values. Based on these findings, the current experiments on 4-inch pipe were conducted with a reduced test matrix spanning *AR* of 1.0 to 2.0 (1, 1.2, … 2.0 for a total of six *AR* values). This range was selected to provide stable estimates of circumferential residual stresses with variability below 10% and while maintaining a conservative sampling approach for the slitting method.

Since evaluating residual stresses by stress relief necessarily requires cutting the specimen and disturbing its initial equilibrium, the possibility of introducing local cutting-induced disturbances must be considered. In the present study, a cutting procedure was devised that minimizes such effects as much as practicable. In contrast to more elaborate slot-milling [10,20] or progressive layer-removal procedures [23], the adopted method requires only two vertical slits and removal of a limited sector from the thin-walled pipes. Cutting was performed with a band saw blade speed that is considerably slower than typical milling operations. Hence, in combination with plentiful coolant assistance, and the polymer being a comparatively poor thermal conductor, heat input into the material is effectively diminished. This process reduced specimen preparation complexity and cutting time while also minimizing thermal and mechanical disturbances during sample preparation. Although cutting-induced effects cannot be assumed to be strictly zero in any sectioning-based method, the implemented procedure was intended to ensure that the measured deformation was governed by release of the pre-existing residual stresses in the pipe wall.

**Figure 1:**
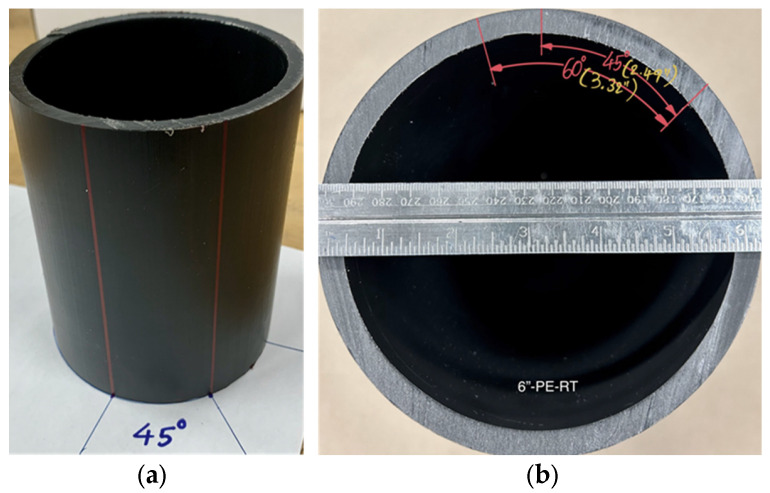
Samples marked for 45° or 60° sectioning (**a**) side-view and (**b**) top-view.

All specimens were cut as adjacent sections from a single pipe using a bandsaw with flowing coolant. Subsequently, a 45° section (for 3-inch pipe) or 60° section (for 4- and 6-inch pipes) was removed from each sample by making simple vertical cuts using a saw with coolant (see Figure 1). This process was shown to be practicable and effective, as it has also been successfully implemented in similar investigations by other researchers [11,13] and in earlier work by the present authors [22].

Once the sectioning of samples was completed, the change in average *OD* of pipe samples was recorded over several days to evaluate circumferential (hoop) residual stresses, *σ*_h_, according to Equation (1), where *D*_0_ is the average *OD* before sectioning and *D_t_* is the average *OD* at given observation time, *t*.

(1) $$\sigma_{h(max)}=\max\left(\pm \frac{E_{t}T}{1-\mu }\times \frac{D_{t}-D_{0}}{D_{t}D_{0}}\right),$$

where *µ* is Poisson’s ratio, *T* is the mean wall thickness of these pipes, and *E_t_* is the apparent modulus at time *t*. The latter will be explained in the subsequent paragraph.

**Figure 2:**
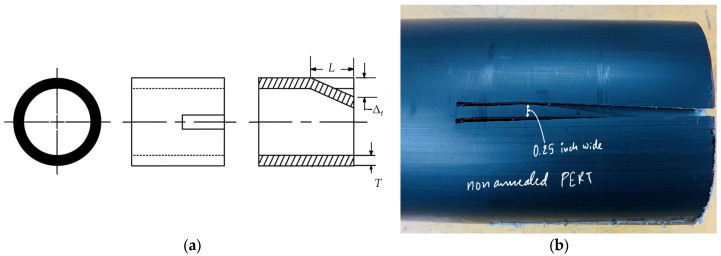
(**a**) Schematics of slitting for determining the longitudinal residual stress (reproduced from [13,22]), and (**b**) 3-inch PE-RT pipe sample tongue-like section.

For assessing the longitudinal residual stresses, *σ*_l_, in these pipes, a tongue-like section was cut lengthwise into the samples as shown in Figure 2. By observing the deflection, Δ*_t_* of the cut section at the free-end over a period of time, *σ*_l_ was evaluated for all the samples using Equation (2).

(2) $$\sigma_{l\left(max\right)}=max\left(\pm \frac{E_{t}\cdot T\cdot \Delta_{t}}{L^{2}}\right),$$

where *L* is the length of longitudinal cut section (see Figure 2a).

PE-RT as a polyethylene pipe material with viscoelastic characteristics, exhibits time-dependent creep and stress-relaxation behavior [24]. Therefore, after slitting a PE-RT pipe to release its residual stresses, the deformation response is expected to evolve with time due to viscoelastic relaxation. Accordingly, the use of an apparent modulus is considered appropriate for evaluation of residual stresses in PE-RT. Because the ASTM E1928-13 standard was originally developed for metallic materials with a time-independent elastic modulus, Equations (1) and (2) were modified from the standard by replacing the modulus of elasticity (*E*) with the creep or apparent modulus (*E_t_*) to account for the viscoelastic nature of the PE-RT material when evaluating the residual stresses. A similar approach was implemented in other studies [13,25]. The apparent modulus values of PE-RT were obtained from tabulated data in the Plastics Pipe Institute handbook [26]. These values were validated through comparison with creep modulus data from an independent study by Choi and Broutman [13], which showed good agreement. Therefore, the handbook values were directly utilized in this experimental study.

Recognizing that the relaxation of tested PE-RT pipe samples is a time-dependent process due to the inherent viscoelastic nature of the material, deformations after slitting were observed for a period of approximately 3 weeks, for which the corresponding residual stresses were evaluated. Once the samples reached a steady state (i.e., no further dimensional changes were observed), the maximum value among all evaluated residual stresses (across all aspect ratios and observation times) was selected as the representative ‘maximum residual stress’ for the material. Residual stresses are undesirable for the intended application of these pipes, as they can promote crack growth or accelerate material failure. Consequently, it is appropriate to report the maximum observed residual stress and pursue strategies for its mitigation.

### 2.2. Annealing of PE-RT Pipe

To investigate mitigation of residual stresses via heat treatment, annealing was performed in a large-scale industrial electrical convection oven using a consistent procedure for all specimens. As-received pipes with a length of 3 m were wrapped in aluminum foil to limit oxidative effects during heating and to promote uniform thermal exposure at temperatures as high as 115 °C. The long length of the sections is justified to support best practice in residual stress evaluation, whereby test samples are extracted from adjacent locations along the same pipe to minimize variability. Using a single, sufficiently long pipe section also avoids variations associated with annealing small, separately cut specimens and ensures adequate material for all residual-stress measurements. Details of the selected annealing temperatures and the corresponding dimensional changes induced by heat treatment are provided in Section 3.3.

### 2.3. X-Ray Diffraction Analysis

**Figure 3:**
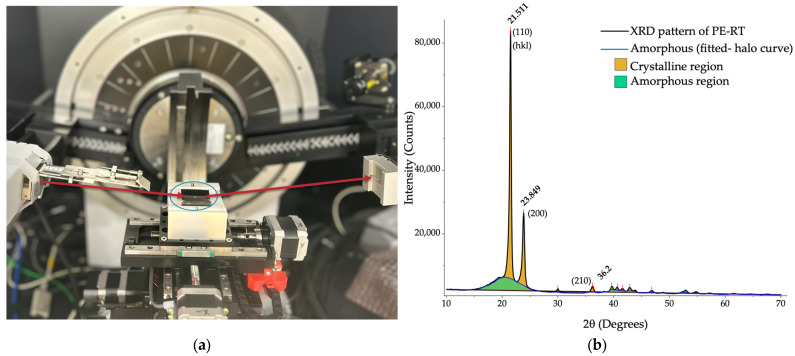
(**a**) XRD diffractometer with the X-ray beam and PE-RT sample indicated by red arrows and a blue ellipse, respectively. (**b**) Typical WAXD pattern of PE-RT pipe samples.

X-ray diffraction (XRD) analysis was performed using a Bruker D8 Discover diffractometer (Billerica, MA, USA) to obtain the wide-angle X-ray diffraction (WAXD) pattern of each of the PE-RT pipe samples in order to evaluate the differences in crystallinity between the inner and outer pipe wall. The objective of performing XRD measurements was to explore whether a correlation exists between the differences in crystallinity between the inner and outer pipe wall and residual stresses mitigation through heat treatment. An incident X-ray beam with a partially collimated size of 1 mm from a Cu-Kα source and a wavelength of 1.54060 Å (0.15406 nm) was directed at the sample as shown in the photograph in Figure 3a. The diffracted beam was collected by the detector as indicated by the red arrows in the figure. The sample was scanned at angles ranging from two-theta (2*θ*) of 10° to 70° to account for all relevant crystalline regions. For consistency, scan parameters were kept identical for all samples. Due to the inability to access both the inner and outer wall with the X-ray beam using ring samples, small square coupons were cut to a size of approximately 25 mm.

As an example, a WAXD pattern for a PE-RT pipe sample is shown in Figure 3b. In this graph, distinctive peaks highlighted in yellow identify the crystalline regions present in the material, while the broad area shaded in green color represents the amorphous phase. According to Bragg’s law, when an incident X-ray beam interacts with regularly spaced lattice planes in a crystalline material, constructive interference occurs at specific diffraction angles, resulting in high-intensity peaks. These sharp peaks therefore indicate the presence of ordered crystalline structures, whereas the diffuse background arises from the amorphous fraction. The degree of crystallinity (%) can be determined by calculating the ratio of the integrated area of the crystalline peaks to the total area under the diffraction curve (crystalline plus amorphous contributions).

## 3. Results and Discussion

### 3.1. Circumferential Residual Stresses

**Figure 4:**
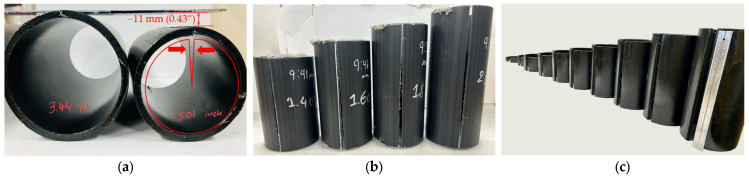
Deformation behavior of as-received PE-RT pipe samples following sectioning for circumferential residual stress evaluation: (**a**) 3-inch sample prior to slitting and after complete relaxation and gap-closure; (**b**) 4-inch samples with different aspect ratios showing similar deformation post-slitting; and (**c**) 6-inch samples displaying gap-closure before complete relaxation.

Figure 4 illustrates the behavior of baseline PE-RT pipes (without annealing) after removing a 45° and 60° section for 3-inch and 4-/6-inch pipe samples, respectively. As shown in Figure 4a, the samples consistently exhibited gap closure (reduction in slit width), indicating the presence of residual stresses manifested through these deformations. Specifically, the observed gap closure after slitting indicates that the as-extruded pipes experience tensile residual stresses on the inner wall, and conversely, compressive residual stress on the outer wall. It should be noted that the as-extruded pipe, as a whole, remains globally stress-free, implying that these residual stresses are in equilibrium across the wall thickness. Disturbing this equilibrium by slitting results in stress relaxation and thus provides the foundation for quantifying residual stresses using the slitting method. As shown in Figure 4a, a change in the *OD* after sectioning was observed over a period of two to three weeks until no further dimensional change was observed (steady state), upon which the maximum circumferential residual stress was evaluated by computing and examining all the residual stress values at the various times of observation.

#### 3.1.1. Circumferential Residual Stresses in 3-Inch Pipes

As per the methodology described above, the circumferential residual stresses were evaluated for the as-received (or baseline) 3-inch pipe by utilizing samples with 12 different *AR* values ranging from 0.2 to 3.0. Deformations in terms of gap closure after slitting were observed to be substantial, suggesting the presence of considerable residual stresses. For example, after 3 h of removing the 45° sections, the gap size reduced to produce *OD* values of 77.98 mm and 78.11 mm for the *AR* values of 1.4 and 3.0, respectively, which corresponds to an approximate reduction in *OD* of 11%.

Compiling the circumferential residual stress, *σ*_h_, for all *AR* values and observation times, the *σ*_h(max)_ for the baseline 3-inch pipes was found to be 6.66 MPa. Notably, this value amounts to about 28% of the material’s yield strength (24.1 MPa). This observation is significant as in the presence of stress risers such as surface imperfections (scratches, gauges), the observed residual stresses may accelerate damage progression, such as crack growth [14,27]. These findings highlight the significance of implementing measures for residual-stress mitigation. Notably, differences in circumferential residual stresses were negligible for samples with *AR* values higher than 2.0. Consequently, *AR* values greater than 2.0 were excluded from the remainder of the study.

It is important to note that although the non-annealed samples appeared to fully close the slit gap after more than two weeks of relaxation, this behavior does not compromise the determination of the maximum circumferential residual stress. The apparent (creep) modulus of PE-RT decreases significantly with time [13,26], and consequently the calculated residual stresses reduce as relaxation progresses. After approximately three weeks of observation, even if additional deformation were to occur, the corresponding stress values would be lower than those recorded during the earlier stages of the experiment. Therefore, the maximum circumferential residual stress is reliably captured within the initial observation period.

#### 3.1.2. Circumferential Residual Stresses in 4- and 6-Inch Pipes

The slitting methodology involving the removal of a 60° section was employed for the 4- and 6-inch pipes, using 10 samples for each nominal size with *AR* values ranging from 0.2 to 2.0. Since the different nominal pipe sizes were extruded in the same manufacturing setting, i.e., pipes were quenched on the outside immediately after extrusion, leaving the interior at higher temperature, similar behavior was expected for the larger nominal pipe sizes. Upon computing the residual stress values for the 4- and 6- inch pipe samples for the different *AR* values and observation times, *σ*_h(max)_ values of 7.45 MPa and 8.33 MPa were obtained, respectively. Indeed, these values are greater than those observed for the 3-inch pipe samples, likely due to differences in the extrusion process, such as the speed of extrusion and the amount of quenching applied. For convenience, the *σ*_h(max)_ values for the different nominal pipe dimensions are tabulated in Table 2. Notably, the higher *σ*_h(max)_ values for the 4- and 6-inch pipe correspond to approximately 31% and 35% of the material’s yield strength (see Table 2), raising even greater concern regarding sensitivity to damage progression from surface imperfections. Risks of pipe damage and failure may increase even further in low-temperature environments, as the material becomes brittle, leading to increased risk of rapid crack propagation, especially for thicker pipe walls [27].

Another noteworthy observation made during these tests was that the gap closures resulted in practically perfect circular conditions, as verified by outer micrometer measurements. This response corroborates the pure bending theory that serves as foundation for deriving Equation (1).

**Table 2:** Maximum circumferential residual stress values, *σ*_h(max)_, for 3-, 4- and 6-inch nominal sizes of as-received PE-RT pipe.

Nominal Pipe Size	*σ*_h(max)_, MPa	*σ*_h(max)_ as Percentage of Yield Strength (24.1 MPa), %
3-inch	6.66	27.6
4-inch	7.45	30.9
6-inch	8.33	34.6

### 3.2. Longitudinal Residual Stresses in 3-, 4- and 6-Inch PE-RT Pipes

Longitudinal residual stresses were determined using Equation (2) based on the deformation of the tongue-like section cut lengthwise into the samples. As shown in Table 3, *σ*_l(max)_ values were found to be lower in magnitude compared to corresponding circumferential data for all three nominal pipe sizes.

**Figure 5:**
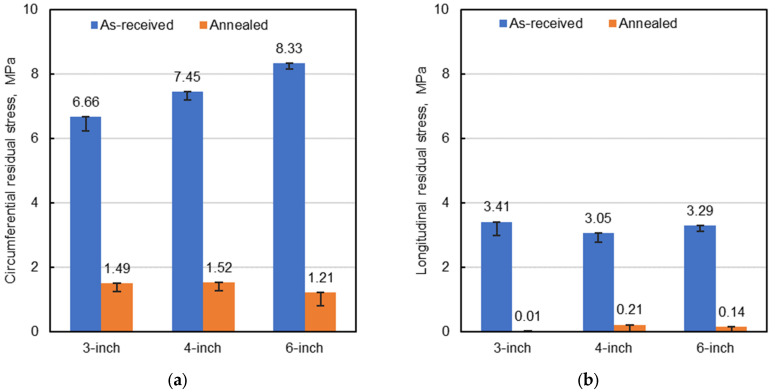
(**a**,**b**) Comparison of residual stresses between as-received PE-RT pipes and samples annealed at 115 °C for 3 h. Error bars indicate the range of the lowest and highest maximum residual stress values observed during the observation period for the considered *AR* range of 1.0 to 2.0.

The data from Table 2 and Table 3 are summarized in Figure 5, which also indicates the range of the lowest and highest maximum residual stress values observed during the observation period for the considered AR range of 1.0 to 2.0.

**Figure 6:**
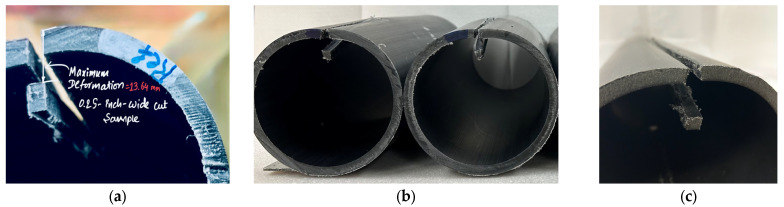
Deformation behavior of as-received PE-RT pipe samples following slitting for longitudinal residual stress evaluation. Images were captured after reaching steady-state deformation: (**a**) 3-inch, (**b**) 4-inch, and (**c**) 6-inch pipe sample.

With regard to the physical appearance of the slit sections, it was observed that all tested samples deformed inward, as shown in Figure 6. This behavior implies that the inner portion of the pipes experienced tensile residual stresses, while the outer portion was subjected to compressive residual stresses, consistent with the observations made for circumferential residual stresses. To reiterate, the material as a whole remains globally stress-free; however, when the equilibrium state of the pipe is disturbed by slitting, the resulting deformation reveals the presence of residual stresses.

Comparing the data in Table 2 and Table 3 (or Figure 5), it is evident that the longitudinal residual stresses are approximately half or less than the corresponding circumferential residual stresses. Several factors may contribute to these differences, with the most predominant being (i) differential cooling resulting from quenching only the outer surface of the pipe and (ii) anisotropic material behavior caused by molecular orientation or alignment in the extrusion direction. Huang et al., 2012, and Nie and Wang, 2013, [28,29] attributed the lower *σ*_l(max)_ values to the organized alignment of molecular chains in the extrusion direction, which promotes improved lamellar orientation and consequently reduced residual stresses along the longitudinal direction. Guevara-Morales and Leevers [6] explained that the lower *σ*_l(max)_ values are a consequence of sudden quenching of the outer surface, which solidifies before the inner layer and leads to differential thermal contraction. This process generates tensile hoop stresses on the inner surface and compressive stresses on the outer surface. While the present observations are largely consistent with these prior studies, further investigation of molecular orientation, for example using grazing-incidence small-angle X-ray scattering (GI-SAXS), could provide additional insight into the anisotropic structural contributions to residual stress development.

### 3.3. Effect of Annealing PE-RT Pipes and Their Benefits

During annealing, careful selection of temperature and dwell time is required to allow the polymer microstructure to undergo beneficial modifications that may lead to an improved degree of crystallinity and mechanical properties. However, identifying an appropriate annealing temperature based on the literature proved inconclusive. Previous studies reported a range of annealing temperatures, and only limited information is available specifically for this grade of polyethylene pipe.

Therefore, to identify an annealing temperature that would result in the lowest possible residual stresses while promoting improved crystallinity, a methodical approach was adopted, beginning with an initial annealing temperature of 90 °C. Considering the significant time required for quantification and comparison of residual stresses following annealing, this sensitivity study was initially conducted using only the 3-inch pipes. The resulting best annealing temperature was subsequently applied to the investigation of the 4- and 6-inch pipes.

Pipe sections of 3 m in length were annealed at 90, 105, and 115 °C for 3 h. Notably, 115 °C approaches the material melting temperature of approximately 130 °C. Further temperature increases would risk excessive material softening during annealing. Therefore, annealing at 115 °C for 3 h provided the most favorable balance between stress relaxation and material stability.

Following heat treatment, the pipes exhibited dimensional changes in *OD* and length of approximately 0.6% and 1.6%, respectively. Circumferential and longitudinal residual stresses were subsequently evaluated using the slitting methods described above. *σ*_h(max)_ values of 3.43, 2.69, and 1.49 MPa were determined for the 90, 105 and 115 °C annealing temperatures, respectively. Compared with the baseline value of *σ*_h(max)_ = 6.66 MPa for the as-received pipe, annealing resulted in substantial reductions of 48, 60, and 78%, respectively. Similarly, *σ*_l(max)_ values were determined as 1.95 and 1.14 MPa for annealing at 90 and 105 °C, respectively, while longitudinal residual stress was effectively eliminated for 115 °C. Compared to the *σ*_l(max)_ value for the as-received pipe (3.41 MPa), reductions of 43, 67, and nearly 100% were achieved for the three annealing temperatures, respectively. These results demonstrate the strong sensitivity of residual stress mitigation to annealing temperature selection, with annealing at 115 °C for 3 h providing the most favorable balance between stress relaxation and material stability. The relaxation of residual stresses during heat treatment is hypothesized to be related to changes in polymer crystallinity and rearrangement of molecular chains, which were further investigated using XRD analysis as described in a subsequent section.

**Figure 7:**
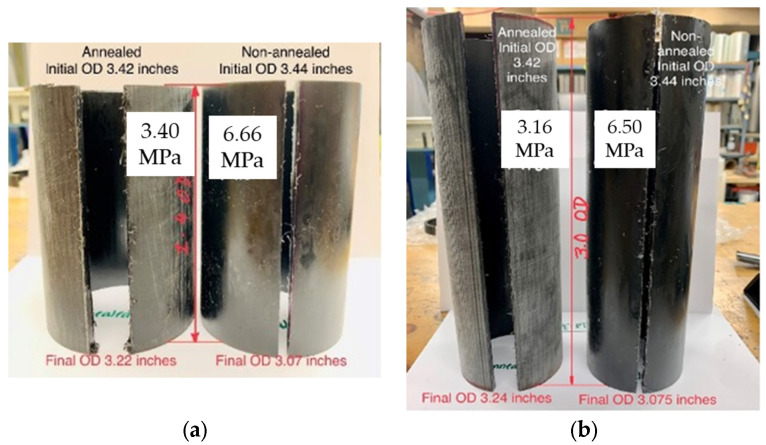
Comparison of final gap-width closure 3 h after cutting for 3-inch PE-RT pipe samples in the as-received condition and after annealing at 90 °C for 3 h, for aspect ratios of (**a**) 1.4 and (**b**) 3.0.

**Figure 8:**
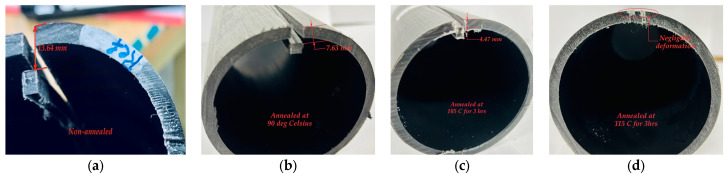
Final deformation of tongue-like sections cut lengthwise into 3-inch PE-RT pipe samples: (**a**) baseline (as-received) and annealed for 3 h at temperatures of (**b**) 90 °C, (**c**) 105 °C, and (**d**) 115 °C.

The photographs in Figure 7 illustrate the reduction in circumferential residual stress resulting from annealing. 3 h after cutting, a much smaller gap-width can be observed for the as-received 3-inch pipes shown on the right of the photos as compared to the samples annealed at 90 °C for 3 h shown on the left. Similarly, Figure 8 illustrates the reduced final deformations of the tongue-like section cut lengthwise into the samples, which indicates a reduction in longitudinal residual stresses following annealing.

As mentioned above, annealing of the 4- and 6-inch samples was performed at 115 °C for 3 h. Residual stresses were determined as previously described, and the resulting values are compared in Table 4 with the corresponding baseline values (i.e., without annealing). While annealing substantially reduced residual stresses in both the circumferential and longitudinal directions for all nominal pipe sizes (78% to ~100%), the percentage reduction varies with pipe size and orientation.

These differences are likely attributable to variations in pipe dimensions and fabrication conditions. For example, the extrusion speed was approximately 9 m/min for 3-inch pipes compared with 3 m/min for 6-inch pipes, which influences cooling conditions, including cooling duration and surface exposure during water quenching. Consequently, residual stresses may develop differently across pipes of different sizes. Notably, despite the substantial residual stress reductions achieved by annealing at 115 °C for 3 h, implementing such heat treatment processes in a manufacturing plant would require considerable economic resources due to the associated energy consumption, processing space requirements, and equipment and maintenance costs.

### 3.4. Measurement of Crystallinity Using XRD

**Figure 9:**
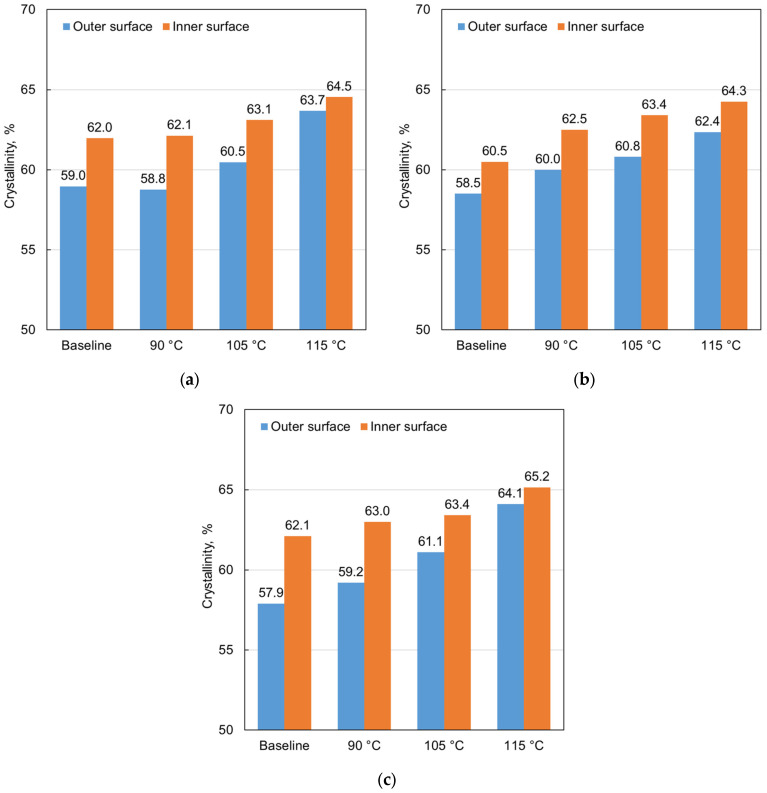
Percent crystallinity of the outer and inner surface of extruded PE-RT pipe with nominal dimensions of (**a**) 3-inch, (**b**) 4-inch, and (**c**) 6-inch, for as-received samples and samples annealed for 3 h at 90 °C, 105 °C, and 115 °C.

To further examine the underlying microstructural mechanisms responsible for the observed stress relaxation during annealing, XRD analysis was performed. It was previously hypothesized that quenching during pipe fabrication causes a rapid temperature reduction at the outer surface of the extruded pipe, thereby suppressing the development of a crystalline polymer phase. Conversely, due to the comparatively low thermal conductivity of the polymer material, this thermal shock is much less pronounced at the inner portion of the pipe, allowing greater polymer crystallinity to develop. These differences in polymer morphology are considered indicative and possibly responsible for the development of compressive residual stresses at the outer portion of the pipe and balancing tensile residual stresses at the inner portion. Furthermore, it is expected that differences in crystallinity between the outer and inner pipe walls diminish when extruded pipes are subjected to annealing. To investigate these hypotheses, XRD testing was performed to measure the degree of polymer crystallinity. Figure 9 presents the percent crystallinity determined via XRD analysis for the outer and inner surfaces of extruded PE-RT pipes of the three nominal dimensions, for both as-received samples and samples annealed for 3 h at 90 °C, 105 °C, and 115 °C.

It can be observed from the data in Figure 9 that, for the as-received pipes of all nominal sizes, the percent crystallinity was lower at the outer pipe wall compared with the pipe interior. The greatest difference of 4.2 percentage points occurred in the 6-inch pipe (i.e., 57.9% and 62.1% at the outer and inner wall, respectively), which can reasonably be attributed largely to the greater wall thickness of this pipe size.

With increasing annealing temperature, the percent crystallinity increased for all pipe sizes. Crystallinity increased at both the outer and inner walls; however, the increase was more pronounced at the outer wall, leading to a reduction in the crystallinity difference between the outer and inner walls. Referring again to the 6-inch pipe, the difference was reduced to only 1.1 percentage points after annealing at 115 °C for 3 h.

The observations from the crystallinity analysis support the interpretation that annealing enables rearrangement of the polymer molecular structure into a more favorable state than in the as-extruded pipe, contributing to both reduced residual stresses and increased crystallinity.

**Figure 10:**
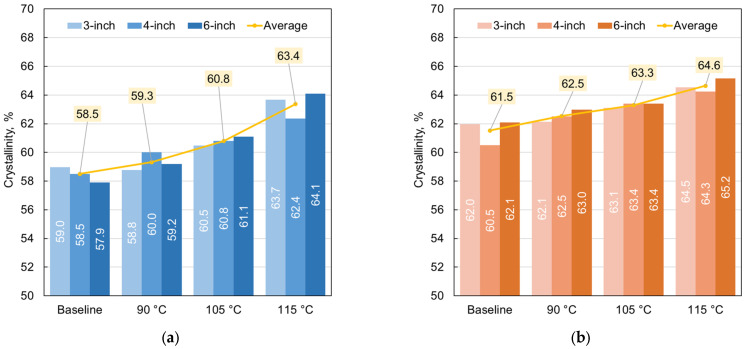
Percent crystallinity of the (**a**) outer and (**b**) inner surface of extruded PE-RT pipe with nominal dimensions of 3-inch, 4-inch, and 6-inch, for as-received samples and samples annealed for 3 h at 90 °C, 105 °C, and 115 °C.

The crystallinity data shown in Figure 9 were reorganized in Figure 10 to separate the results for the outer and inner pipe walls. In addition, an average crystallinity value was calculated for each treatment condition for both cases. The graphs again indicate an overall increase in crystallinity with increasing annealing temperature compared with the baseline values, with a more pronounced rise observed for the outer pipe walls.

Interestingly, the individual values contributing to the mean lie within approximately one percentage point of the respective average. This close agreement in behavior is noteworthy since all nominal pipe sizes share the same SDR of 17. This observation suggests that findings for pipes with a certain SDR may be extrapolated to estimate crystallinity for pipes of different nominal dimensions but the same SDR, provided that fabrication conditions are sufficiently similar.

## 4. Conclusions

In this study, a systematic investigation was conducted to quantify the residual stresses developed during extrusion of three different sizes of PE-RT pipes. Particular focus was placed on establishing the slitting method as an experimental technique for assessing residual stresses in both the circumferential and longitudinal pipe directions. Furthermore, the sensitivity of residual stresses to annealing at elevated temperatures was investigated. In this context, variations in crystallinity were measured using XRD analysis. The study explored the hypothesis that residual stresses arise from differences in polymer morphology between the outer and inner pipe walls caused by rapid quenching of the outer pipe surface during extrusion, and that annealing at elevated temperatures reduces these morphological differences and, consequently, the associated residual stresses, as indicated by a more uniform degree of crystallinity across the pipe wall.

The experiments using the slitting method confirmed the existence of residual stresses (i.e., tensile at the inner portion and compressive at the outer portion) for all pipe sizes, which is consistent with the aforementioned hypothesis. It was found that the maximum residual stresses (i.e., circumferential direction: 6.66, 7.45, and 8.33 MPa for 3-, 4-, and 6-inch pipes, respectively) were significant compared with the material’s yield strength (24.1 MPa) and are therefore undesirable for service applications. Residual stresses were significantly reduced by annealing the pipes at 115 °C for 3 h; circumferential residual stresses were reduced by 78%, 80%, and 85% for 3-, 4-, and 6-inch pipes, respectively, while longitudinal residual stresses were nearly eliminated.

It should be noted that this investigation was conducted using PE-RT pipes obtained from a single manufacturer; therefore, the absolute residual-stress values reported here should not be interpreted as universally representative of all PE-RT pipes, since differences in resin source and processing history may influence the resulting residual-stress state. Nevertheless, the results provide useful insight into the role of pipe size and processing-related conditions within a controlled manufacturing context.

Crystallinity analysis indicated that the inner pipe wall exhibited higher crystallinity compared with the outer wall, which is consistent with the stated hypothesis. It was further observed that after annealing at 115 °C for 3 h, crystallinity increased overall but to a greater extent at the outer wall, leading to a substantially reduced crystallinity gradient across the pipe wall.

The experimental techniques presented in this work offer a suitable framework for evaluating residual stresses in extruded thermoplastic polymer pipes. These approaches may support further research and quality control efforts aimed at optimizing pipe manufacturing processes. Nevertheless, it is acknowledged that residual stress mitigation via annealing involves considerable costs in terms of energy consumption, additional processing facilities, and equipment maintenance. Therefore, future work should investigate alternative processing approaches that reduce residual stresses in extruded PE-RT pipes without requiring elevated-temperature heat treatment.

## Figures and Tables

**Table 3:** Maximum longitudinal residual stress values, *σ*_l(max)_, for 3-, 4- and 6-inch nominal sizes of as-received PE-RT pipe.

Nominal Pipe Size	*σ*_l(max)_, MPa	*σ*_l(max)_ as Percentage of Yield Strength (24.1 MPa), %
3-inch	3.41	14.1
4-inch	3.05	12.7
6-inch	3.29	13.7

**Table 4:** Comparison of residual stresses between as-received PE-RT pipes and samples annealed at 115 °C for 3 h.

Nominal Pipe Size	Maximum Circumferential Residual Stress, *σ*_h(max)_			Maximum Longitudinal Residual Stress, *σ*_l(max)_		
	Baseline, MPa	After Annealing, MPa	Percent Reduction, %	Baseline	After Annealing, MPa	Percent Reduction, %
3-inch	6.66	1.49	78	3.41	0.01	~100
4-inch	7.45	1.52	80	3.05	0.21	93
6-inch	8.33	1.21	85	3.29	0.14	96

## Data Availability

The original contributions presented in this study are included in the article. Further inquiries can be directed to the corresponding author.
